# TREM2 deficiency exacerbates cognitive impairment by aggravating α-Synuclein-induced lysosomal dysfunction in Parkinson’s disease

**DOI:** 10.1038/s41420-025-02538-1

**Published:** 2025-05-20

**Authors:** Baoyu Zhu, Jiezhu Feng, Xiaomei Liang, Zhongling Fu, Mengshi Liao, Tongtong Deng, Kaicheng Wang, Jianwei Xie, Jieshan Chi, Lu Yang, Yuyuan Gao, Kun Nie, Lijuan Wang, Piao Zhang, Yuhu Zhang

**Affiliations:** 1 Department of Neurology, Guangdong Neuroscience Institute, Guangdong Provincial People’s Hospital (Guangdong Academy of Medical Sciences), Southern Medical University, Guangzhou, Guangdong Province China; 2 Guangzhou Key Laboratory of Diagnosis and Treatment for Neurodegenerative Diseases, Guangdong Provincial People’s Hospital (Guangdong Academy of Medical Sciences), Guangzhou, China; 3 School of Medicine, South China University of Technology, Guangzhou, Guangdong Province China; 4 Department of Neurology, Longyan First Hospital, Fujian Province, China

**Keywords:** Parkinson's disease, Apoptosis

## Abstract

Cognitive impairment in Parkinson’s disease (PD) is a widespread and rapidly progressive feature that impacts prognosis. Although TREM2 has been implicated in neuroprotection across various neurodegenerative diseases, its specific role in PD remains to be clarified. In this study, we first detected the hippocampus of human PD specimens and of the mutant A53T α-Synuclein transgenic mice (A53T mice), and found a significant increase in the number of TREM2^+^ microglia. To evaluate the effects of TREM2 deficiency, TREM2-deficient A53T mice (TREM2^-/-^/A53T mice) were generated. In these mice, exacerbated cognitive impairment, neurodegeneration, disruption of synaptic plasticity, and accumulation of pathological α-Synuclein (α-Syn) in the hippocampus were observed, without any detected motor dysfunction. Despite increased infiltration of activated microglia surrounding α-Syn aggregates, lysosomal dysfunction in microglia was aggravated in the TREM2^-/-^/A53T mice. In addition, transcriptional analyses and in vitro experiments further found that TREM2 knockdown inhibited the nuclear distribution of TFEB via the ERK1/2 pathway, exacerbating α-Syn-induced lysosomal dysfunction and causing more pathological α-Syn accumulation. Finally, HT22 cells were cocultured with TREM2 knockdown of BV-2 cells pretreated with recombinant human A53T α-Syn preformed fibrils (PFFs). The coculture experiments showed that TREM2 knockdown in BV-2 cells pretreated with PFFs enhanced the phosphorylation of α-Syn and promoted apoptosis in HT22 cells via inhibiting α-Syn degradation. In conclusion, TREM2 deficiency exacerbates cognitive impairment in PD by exacerbating α-Syn-induced microglial lysosomal dysfunction, identifying TREM2 as a potential therapeutic target.

## Introduction

Parkinson’s disease (PD) is a progressively neurodegenerative disease characterized by the loss of dopaminergic neurons in the substantia nigra pars compacta (SNpc) and the formation of Lewy bodies by the misfolded and aggregated α-Synuclein (α-Syn) [1]. The misfolded and aggregated α-Syn propagates in a prion-like manner [2], causing neuronal damage [3]. While PD is traditionally associated with motor dysfunction, cognitive impairment is increasingly recognized as an early and progressive feature that significantly impacts prognosis [4]. Pathologically, misfolded α-Syn in cognitive-related brain regions, particularly the hippocampus, is a key contributor to cognitive impairment in PD [5].

TREM2 is a type of trans-membrane protein containing V-type Immunoglobulin (Ig) domain [6]. In the central nervous system, TREM2 is mainly expressed in microglia, which have been shown to clear the misfolded and aggregated α-Syn in PD [7], participating in the proliferation, phagocytosis, survival, and expression of inflammatory factors in microglia [8]. TREM2 has been shown to play a vital role in cognitive-related neuroprotection in multiple diseases, such as nonalcoholic fatty liver disease [9], postoperative cognitive impairment [10], Nasu-Hakola disease [11], vascular dementia [12], and Alzheimer’s disease (AD) [13, 14]. Notably, the TREM2 R47H mutation has been found to be a strong genetic risk factor for AD [13], and TREM2 is involved in synaptic plasticity [15] and cognitive improvement in AD mice [13, 16]. Moreover, TREM2 has been shown to alleviate neurodegeneration and neuroinflammation in the PD mice [17, 18]. However, the role of TREM2 in cognitive impairment in PD remains unclear.

Lysosomes are membrane-enclosed vesicle organelles that contain two types of proteins necessary for maintaining structure and function: soluble lysosomal hydrolases CathB, CathD, and so on that perform digestive functions and lysosomal membrane proteins Lamp1, Lamp2, and so on with more complex functions [19–24]. In microglia, internalized α-Syn were targeted to the lysosomes for degradation [25–28]. While being degraded, α-Syn also destroys lysosomal function in a variety of ways: α-Syn interacts with receptors or proteins on the lysosomal membrane, causing the stability of the lysosomal membrane to decrease, affecting the integrity of the lysosomal membrane, thereby inhibiting the activity of lysosomal enzymes [29, 30], especially in the α-Syn mutant A53T, where it is more likely to occupy the lysosomal binding site with high affinity [31]; α-Syn can also block the lysosomal degradation pathway and interfere with the autophagy-lysosomal pathway [29]. Ultimately, the misfolded and aggregated α-Syn cannot be completely dissolved by the lysosome, inducing lysosomal exocytosis and exacerbating the aggregation and pathological proliferation of α-Syn [26, 32–34]. Multiple studies have demonstrated that lysosome-related genes are associated with TREM2 expression [35–37]. However, lysosomal function exhibits significant variability across different TREM2 knockdown disease models, and the specific changes in lysosomal function in PD remain unclear.

In this study, we knocked out TREM2 in the mutant A53T α-Syn transgenic mice (A53T mice) to investigate the effect of TREM2 deficiency on cognitive impairment in PD mice, as well as synaptic plasticity and the degradation of α-Syn in their hippocampus. We next knocked down TREM2 in BV-2 cells and treated the cells with recombinant human A53T α-Syn preformed fibrils (PFFs) to investigate the effect of knockdown of TREM2 on α-Syn-induced lysosomal dysfunction in microglia. Then, transcriptome analysis of hippocampal tissues revealed that ERK1/2 is a crucial downstream pathway of TREM2 deficiency affecting A53T mice. Results showed that TREM2 deficiency inhibited the nuclear distribution of TFEB via the ERK1/2 pathway, exacerbating α-Syn-induced lysosomal dysfunction and causing more pathological α-Syn accumulation, which could be reversed by ERK1/2 inhibitor. Finally, we cocultured HT22 neuronal cells with TREM2 knockdown in BV-2 cells pretreated with PFFs to investigate the effect of TREM2 deficient microglia on neurons via affecting α-Syn degradation. Our study suggested a potential target for the treatment of cognitive impairment in PD and provides new ideas of animal models for exploring cognitive impairment in PD.

## Results

### TREM2 deficiency impaired cognitive function in PD mice

**Fig. 1:**
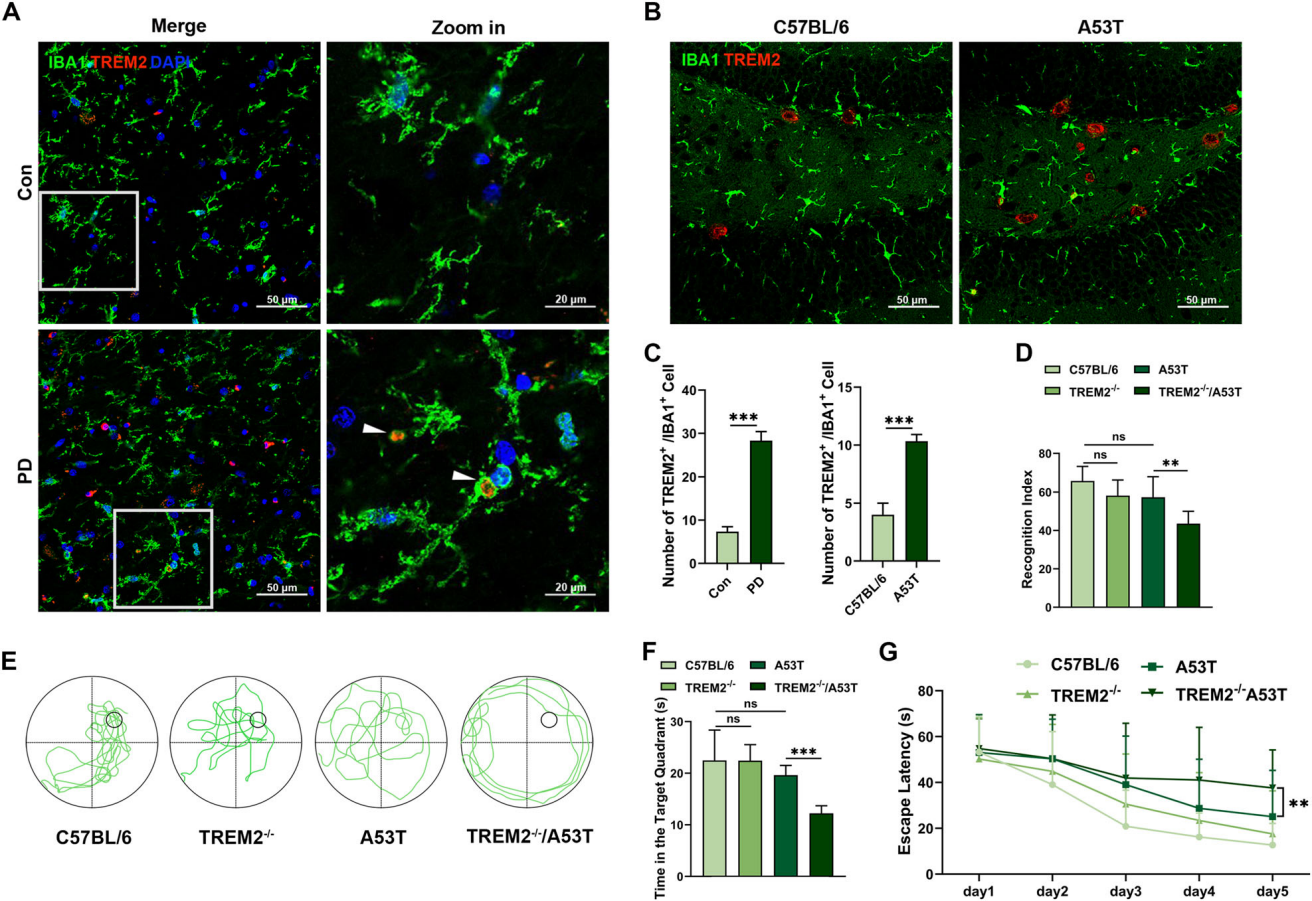
TREM2 deficiency impaired cognitive function in PD mice. **A** Representative image of TREM2 (red) and IBA1 (green) immunofluorescence staining in hippocampal tissue sections of the PD and control specimens. Scale bar, 50μm. **B** Representative image of TREM2 (red) and IBA1 (green) immunofluorescence staining in hippocampus in C57BL/6 mice and A53T mice. Scale bar, 50 μm. **C** Quantification of number of TREM2^+^ IBA1^+^ cells in PD specimens and control specimens (*n* = 3, unpaired Student’s t test) and TREM2^+^ IBA1^+^ cells in C57BL/6 mice and A53T mice (*n* = 3, unpaired Student’s *t* test). **D** Recognition Index was calculated in NORT (*n* = 9, one-way ANOVA and Šídák’s multiple comparisons test). **E** Representative image showing the procedure and swimming paths obtained from each group in the acquisition phase in MWM. **F** Time spent in the target quadrant (s) in the acquisition phase in MWM (*n* = 9, one-way ANOVA and Šídák’s multiple comparisons test). **G** Representative image showing the paths obtained from each group in the acquisition phase in Open field test. Line chart showing the average escape latencies in each group in the space exploring phase in MWM (*n* = 9, two-way ANOVA and Tukey’s multiple comparisons test). Column charts showing. The error bars represent the ± SDs. **p < 0.01, ***p < 0.001. NORT, novel object recognition test; MWM, Morris water maze.

We collected the hippocampal tissues from human cadavers and mice, revealing that the counts of TREM2^+^ microglia in the PD specimens and A53T mice were significantly higher than those in controls (Fig. 1A–C). These results suggested that TREM2 may play a role in the hippocampus of PD.

Then, we performed behavioral tests to explore the effects of TREM2 in PD mice. Novel object recognition test (NORT) revealed that the TREM2^-/-^/A53T mice had lower recognition index than the TREM2^-/-^ and the A53T mice did (Fig. 1D). Morris water maze (MWM) test revealed that, compared with the TREM2^-/-^ and the A53T mice, the TREM2^-/-^/A53T mice showed significantly longer escape latency in the space exploring phase and significantly shorter duration in the target quadrant in the acquisition phase (Fig. 1E–G). Other behavioral tests showed no significant differences in motor function of the mice in each group (Figure S1A–F). In addition, knocking out TREM2 did not affect the number of dopaminergic neurons in mice (Figure S1G–L). These results indicate that TREM2 deficiency exacerbates cognitive deficits in PD without significantly impacting motor symptoms or dopaminergic neuron survival.

### TREM2 deficiency exacerbated α-Syn accumulation in the hippocampus via lysosomal dysfunction

**Fig. 2:**
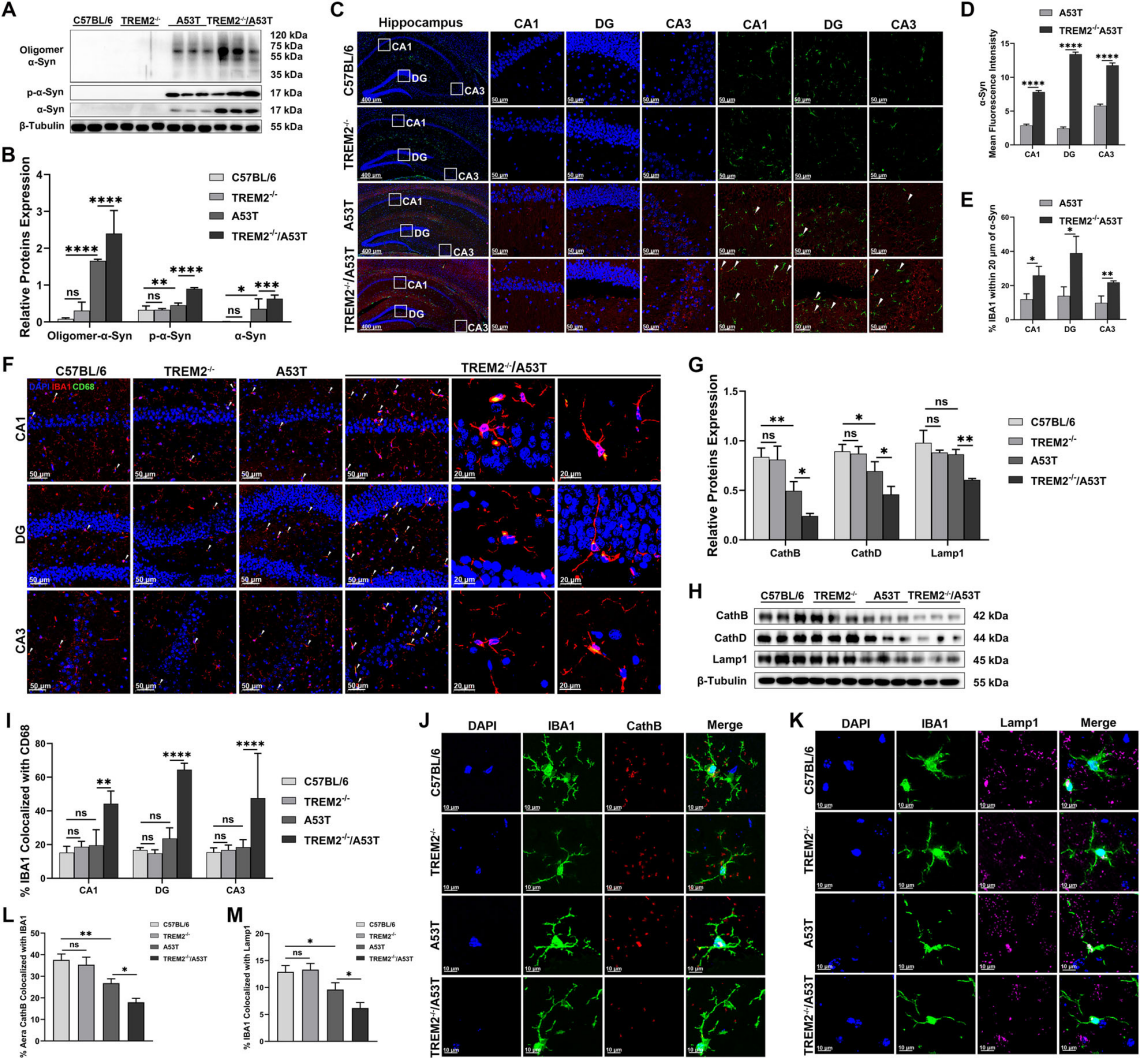
TREM2 deficiency exacerbated α-Syn accumulation and lysosomal dysfunction in the hippocampus. **A** Representative images of western blotting analysis of Oligomer α-Syn, p-α-Syn, and α-Syn expressions in each group. **B** Quantification of Oligomer α-Syn, P-α-Syn, and α-Syn proteins relative expression (*n* = 3, one-way ANOVA and Šídák’s multiple comparisons test). **C** Representative images of α-Syn (red) and IBA1 (green) in the hippocampus of each group. Scale bar, 400μm. **D** Quantification of mean Fluorescence intensity of α-Syn in the hippocampus of each group (*n* = 3, unpaired Student’s *t* test). **E** Quantification of IBA1 within 20 μm of α-Syn (%) in the hippocampus of each group (*n* = 3, unpaired Student’s *t* test). **F** Representative images of IBA1 (red) and CD68 (green) immunofluorescence staining in the hippocampus of each group. Scale bar, 50μm. **G** Quantification of IBA1 colocalized with CD68 (%) in the hippocampus of each group (*n* = 3, one-way ANOVA and Šídák’s multiple comparisons test). **H** Representative images of western blotting analysis of CathB, CathD, and Lamp1 expressions in each group. **I** Quantification of CathB, CathD, and Lamp1 proteins relative expression (*n* = 3, one-way ANOVA and Šídák’s multiple comparisons test). **J** Representative images of CathB (red) and IBA1 (green) immunofluorescence staining in the hippocampus of each group. Scale bar, 10μm. **K** Representative images of Lamp1 (purple) and IBA1 (green) immunofluorescence staining in the hippocampus of each group. Scale bar, 10μm. **L** Quantification of area CathB colocalized with IBA1 (%) in the hippocampus of each group (*n* = 3, one-way ANOVA and Šídák’s multiple comparisons test). **M** Quantification of IBA1 colocalized with Lamp1 (%) in the hippocampus of each group (*n* = 3, one-way ANOVA and Šídák’s multiple comparisons test). The error bars represent the ± SDs. *p < 0.05, **p < 0.01, ***p < 0.001, ****p < 0.0001.

Next, the accumulation of α-Syn in cognitive-related brain regions was examined. Western blotting showed pathological expression of α-Syn in hippocampus in A53T mice. The expression of oligomer α-Syn, p-α-Syn, and α-Syn in the hippocampus (Fig. 2A, B) and cortex (Fig. S2A, B) of TREM2^-/-^/A53T mice was significantly increased, with oligomeric α-Syn and p-α-Syn being primary pathogenic components. Immunofluorescence revealed that the expression of α-Syn in the hippocampal CA1, DG, and CA3 regions in the TREM2^-/-^/A53T mice was significantly greater than those in the A53T mice (Fig. 2C, D). Consistent with α-Syn accumulation, TREM2^-/-^/A53T mice showed significant neuronal damage and synaptic plasticity impairment in hippocampus (Figure S3) and cortex regions (Fig. S2). However, we did not find evidence of significant neuron loss in hippocampal CA1, DG, and CA3 regions in A53T mice despite the pathological aggregation of α-Syn (Figure S2, S3), which was consistent with previous research [38]. Additionally, microglial clustering (IBA1^+^ cells) around α-Syn aggregates was more pronounced in TREM2^-/-^/A53T mice than in A53T mice (Fig. 2C, E); however, infiltrating microglia failed to effectively clear deposited α-Syn. These results suggest that TREM2 deficiency exacerbates α-Syn aggregation due to impaired microglial clearance. However, the CD68^+^ IBA1^+^ cells in the hippocampal CA1, DG, and CA3 regions in the TREM2^-/-^/A53T mice significantly increased (Fig. 2F, G), which indicated the increase in CD68^+^ microglia generally indicates enhanced lysosomal phagocytic activity of microglia, suggesting that the inhibitory effect of TREM2 deficiency on microglial clearance of α-Syn is not achieved by inhibiting its phagocytosis and presentation. Therefore, the study focused on the role of TREM2 deficiency on microglia digestion.

Lysosomes are critical for α-Syn degradation, relying on intact lysosomal membranes and active hydrolytic enzymes. Western blotting showed that the expressions of CathB, CathD (hydrolytic enzymes), and Lamp1 were significantly lower in the A53T mice than those in the C57BL/6 mice and greater than those in the TREM2^-/-^/A53T mice, whereas the expression of CathB, CathD, and Lamp1 in the TREM2^-/-^ mice showed no significant difference compared with the C57BL/6 mice (Fig. 2H, I). The percentage of IBA1 colocalized with Lamp1 and the percentage of area CathB colocalized with IBA1 were decreased significantly in the hippocampus of the TREM2^-/-^/A53T mice compared with the TREM2^-/-^ mice and the A53T mice, compared with the C57BL/6 mice, but there was no difference between the C57BL/6 mice and the TREM2^-/-^ mice (Fig. 2J–M). These results indicated that TREM2 deficiency exacerbated lysosomal membrane permeabilization and hydrolytic enzymes inactivation induced by α-Syn, thereby aggravating lysosomal dysfunction.

### Knockdown of TREM2 aggravated α-Syn-induced lysosomal dysfunction in BV-2 cells

**Fig. 3:**
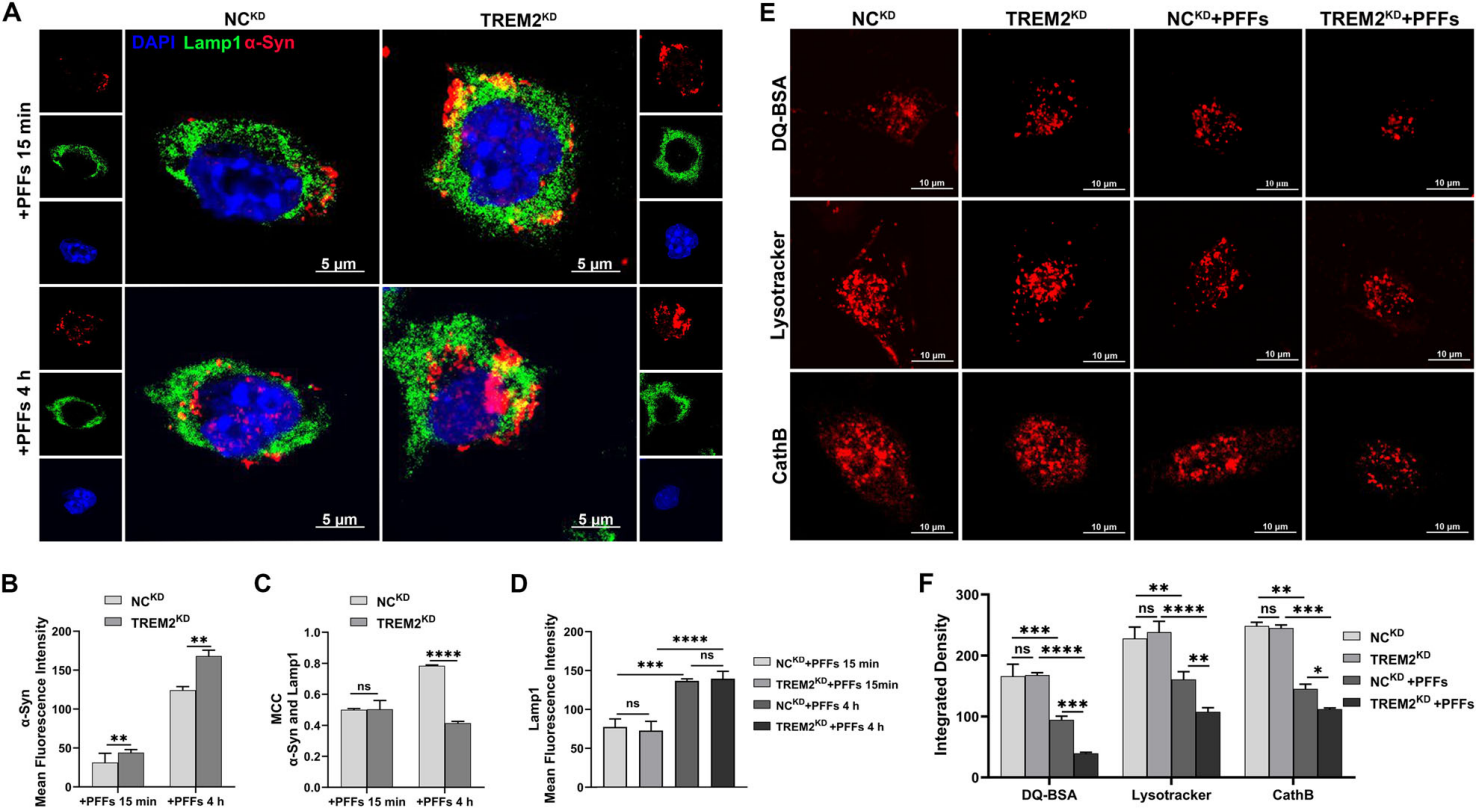
Knockdown of TREM2 exacerbated α-Syn accumulation and lysosomal dysfunction in BV-2 cells. **A** Representative images of α-Syn (red) and Lamp1 (green) immunofluorescence staining in TREM2^KD^ BV-2 cells and NC^KD^ BV-2 cells. Scale bar, 5μm. **B** Quantification of mean Fluorescence intensity of α-Syn in TREM2^KD^ BV-2 cells and NC^KD^ BV-2 cells (*n* = 3, unpaired Student’s t test). **C** Quantification of MCC of α-Syn and Lamp1 in TREM2^KD^ BV-2 cells and NC^KD^ BV-2 cells (*n* = 3, unpaired Student’s t test). **D** Quantification of mean Fluorescence intensity of Lamp1 in TREM2^KD^ BV-2 cells and NC^KD^ BV-2 cells (*n* = 3, one-way ANOVA and Šídák’s multiple comparisons test). **E** Representative images of DQ-BSA (red), Lysotracker (red), and CathB (red) in TREM2^KD^ BV-2 cells and NC^KD^ BV-2 cells. Scale bar, 10μm. **F** Quantification of integrated density of DQ-BSA, Lysotracker, and CathB in TREM2^KD^ BV-2 cells and NC^KD^ BV-2 cells (n = 3, one-way ANOVA and Šídák’s multiple comparisons test). The error bars represent the ± SDs. *p < 0.05, **p < 0.01, ***p < 0.001, ****p < 0.0001. PFFs, recombinant human A53T α-Syn pre-formed fibrils; MCC, Manders’ Colocalization Coefficients; TREM2^KD^ BV-2, BV-2 transfected with TREM2-shRNA, NC^KD^ BV-2, BV-2 transfected with LV-NC; MCC, Manders’ Colocalization Coefficients.

To investigate how TREM2 knockdown affects α-Syn clearance in BV-2 microglial cells, we exposed cells to PFFs. After 15-min PFFs exposure as in a previous study [39], immunofluorescence revealed significantly higher α-Syn levels in the TREM2^KD^ BV-2 cells compared to the NC^KD^ BV-2 cells (Fig. 3A, B). After 4-hour PFFs exposure 4 hours of exposure, the α-Syn in the TREM2^KD^ BV-2 cells was significantly increased and the co-localization of α-Syn with Lamp1 was significantly decreased compared with the NC^KD^ BV-2 cells (Fig. 3A, C). In addition, the abundance of lysosome, measured by mean Lamp1 fluorescence intensity, depended on PFFs exposure time but was unaffected by TREM2 knockdown (Fig. 3A, D). These results suggest that knockdown of TREM2 impaired α-Syn degradation in microglia, independent of lysosomal abundance.

Then we examined the lysosomal activity of BV-2 cells. Before exposure to PFFs, there was no difference in DQ-BSA, Lysotracker DR, and CathB fluorescence intensities between TREM2^KD^ BV-2 cells and NC^KD^ BV-2 cells. After 4 h of exposure to PFFs, the DQ-BSA, Lysotracker DR, and CathB fluorescence intensities were significantly decreased, and knockdown of TREM2 significantly aggravated this decrease (Fig. 3E, F). The results indicated that TREM2 knockdown increased the degradation disorder of PFFs by aggravating the α-Syn-induced damage to lysosomal function. These results indicate that knockdown of TREM2 exacerbated α-Syn-induced lysosomal dysfunction, characterized by disruption of the lysosomal acidic environment and inactivation of lysosomal hydrolases.

### TREM2 regulated the positive regulation of the ERK1 and ERK2 cascade in A53T mice

**Fig. 4:**
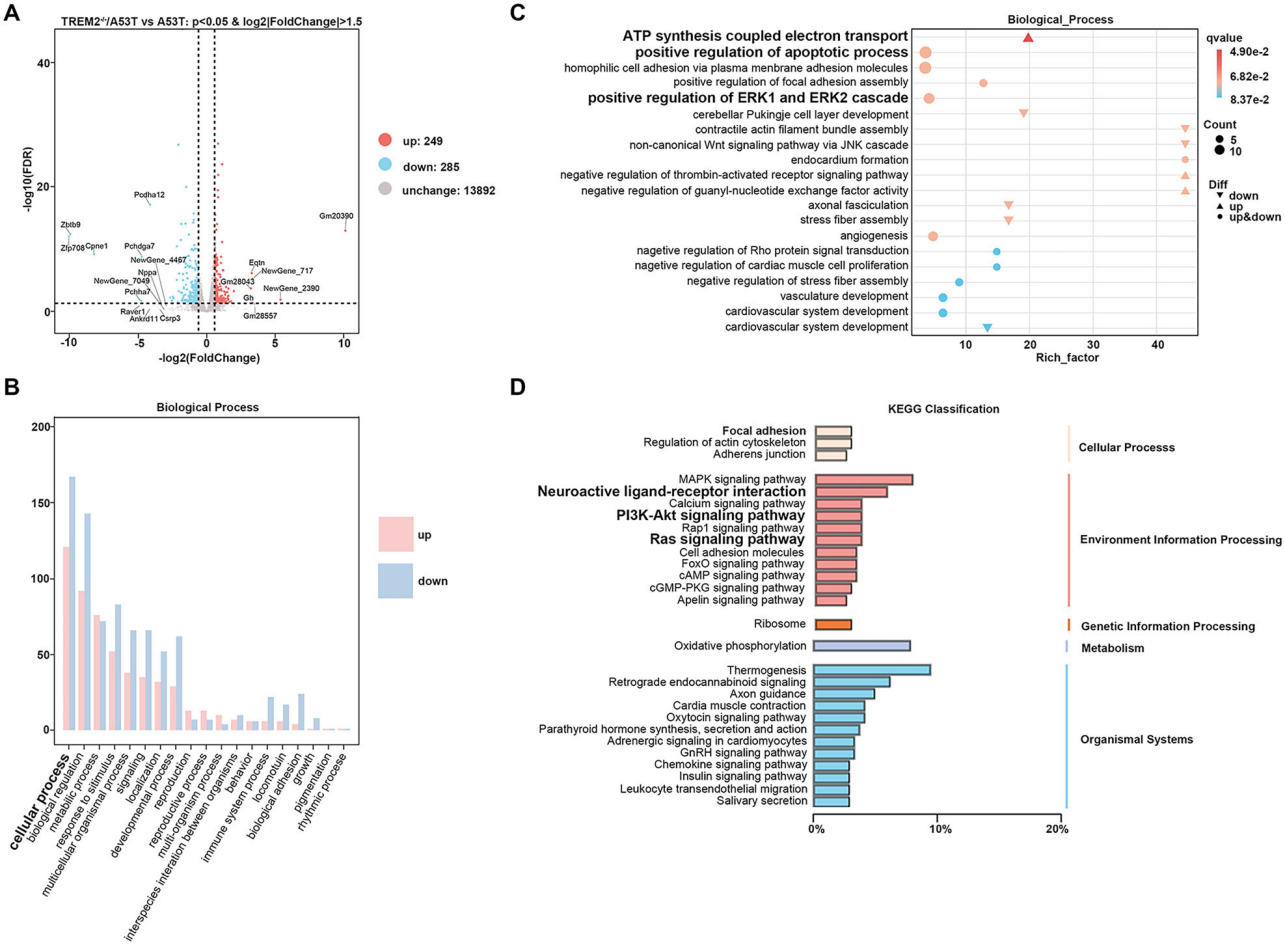
Transcriptome analysis. **A** Volcano plot for differentially expressed genes. **B** GO database on biological process of differentially expressed genes. **C** Identification key pathway by GO of differentially expressed genes. **D** KEGG pathway classification of differentially expressed genes during environment information processing. GO Gene Ontology, KEGG Kyoto Encyclopedia of Genes and Genomes.

Transcriptome analysis was performed to explore the downstream pathways of TREM2 in the hippocampus of PD mice. There was no overall change in mRNA expression distribution. Among the total 534 genes with log_2_|FoldChange|>1.5 and False Discovery Rate (FDR) < 0.05 (Fig. 4A), 249 genes were upregulated and 285 genes were downregulated in the hippocampal tissues of the TREM2^-/-^/A53T mice compared with the A53T mice. After analyzing via the GO database, the results showed that, compared with the A53T mice, the differentially expressed genes in the TREM2^-/-^/A53T mice were heavily concentrated on the positive regulation of the ERK1 and ERK2 cascade (Fig. 4B, C), which has been reported to regulate lysosomal function by regulating TFEB. In addition, analyzing via the KEGG database revealed that the differentially expressed genes in the TREM2^-/-^/A53T mice were heavily concentrated on the PI3K-Akt signaling pathway (Fig. 4D).

### Knockdown of TREM2 aggravated α-Syn-induced lysosomal dysfunction in BV-2 cells via the ERK1/2/TFEB pathway

**Fig. 5:**
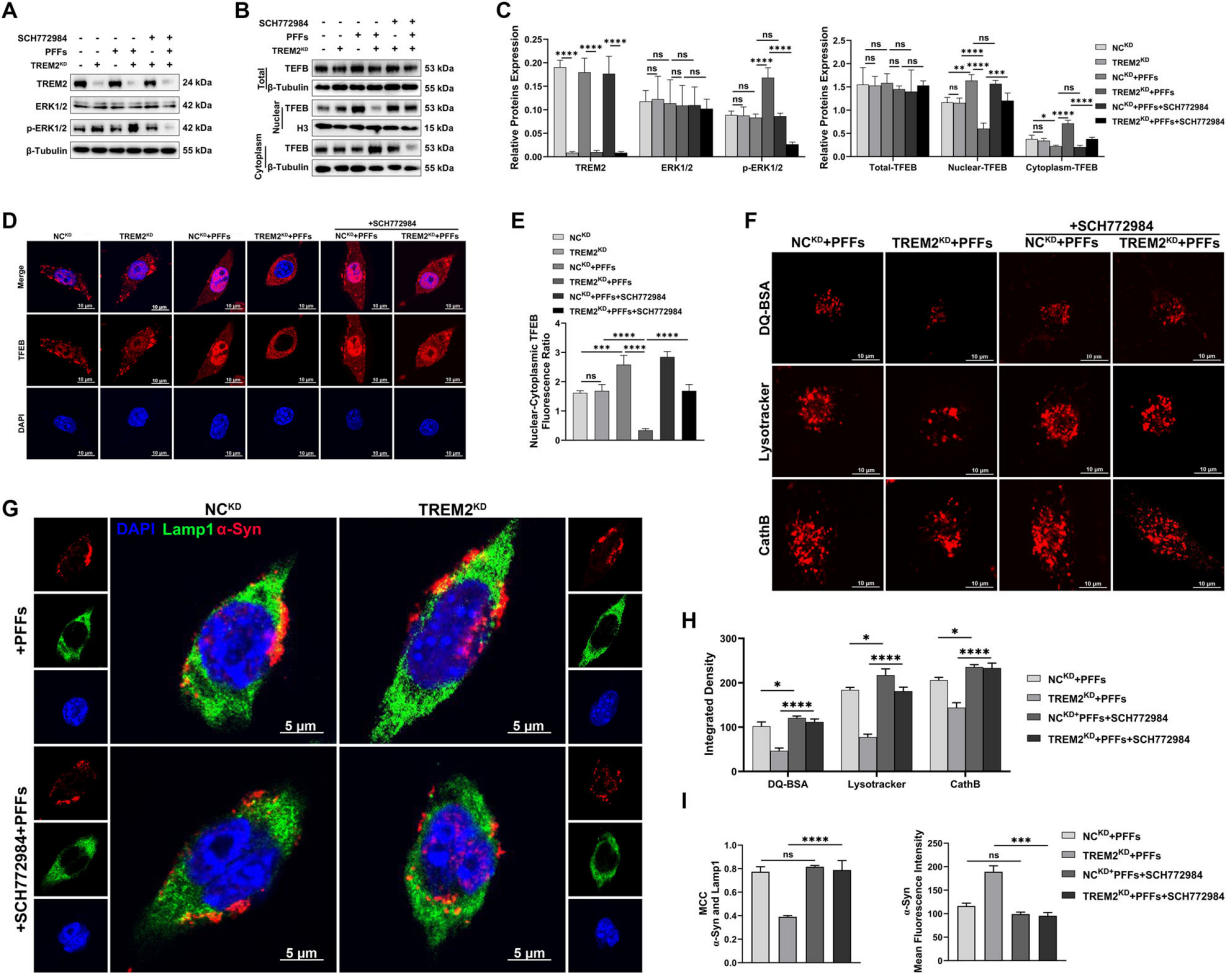
Knockdown of TREM2 aggravated α-Syn-induced lysosomal dysfunction in BV-2 cells via the ERK1/2/TFEB pathway. **A** Representative images of western blotting analysis of TREM2, ERK1/2, and p-ERK1/2 expressions in TREM2^KD^ BV-2 cells and NC^KD^ BV-2 cells. **B** Representative images of western blotting analysis of Total-TFEB, Nuclear-TFEB, and Cytoplasm-TFEB expressions in TREM2^KD^ BV-2 cells and NC^KD^ BV-2 cells. **C** Quantification of TREM2, ERK1/2, p-ERK1/2, Total-TFEB, Nuclear-TFEB, and Cytoplasm-TFEB expressions proteins relative expression (*n* = 3, one-way ANOVA and Šídák’s multiple comparisons test). **D** Representative images of TFEB (red) immunofluorescence staining in TREM2^KD^ BV-2 cells and NC^KD^ BV-2 cells. Scale bar, 10 μm. **E** Quantification of Nuclear- Cytoplasm TFEB Fluorescence ratio in TREM2^KD^ BV-2 cells and NC^KD^ BV-2 cells (*n* = 3, one-way ANOVA and Šídák’s multiple comparisons test). **F** Representative images of DQ-BSA (red), Lysotracker (red), and CathB (red) in TREM2^KD^ BV-2 cells and NC^KD^ BV-2 cells. Scale bar, 10μm. **G** Representative images of α-Syn (red) and Lamp1 (green) immunofluorescence staining in TREM2^KD^ BV-2 cells and NC^KD^ BV-2 cells. Scale bar, 5 μm. **H** Quantification of integrated density of DQ-BSA, Lysotracker, and CathB in TREM2^KD^ BV-2 cells and NC^KD^ BV-2 cells (*n* = 3, one-way ANOVA and Šídák’s multiple comparisons test). **I** Quantification of MCC of α-Syn and Lamp1 in TREM2^KD^ BV-2 cells and NC^KD^ BV-2 cells (*n* = 3, unpaired Student’s *t* test) and mean Fluorescence intensity of α-Syn in TREM2^KD^ BV-2 cells and NC^KD^ BV-2 cells (*n* = 3, unpaired Student’s *t* test). The error bars represent ± SDs. *p < 0.05, **p < 0.01, ***p < 0.001, ****p < 0.0001. PFFs, recombinant human A53T α-Syn pre-formed fibrils; MCC, Manders’ Colocalization Coefficients; TREM2^KD^ BV-2, BV-2 transfected with TREM2-shRNA, NC^KD^ BV-2, BV-2 transfected with LV-NC.

Next, we explored the regulatory role of the ERK1/2 pathway in this pathological process in vitro. Western blotting revealed that the expression of p-ERK1/2 was significantly increased in TREM2^KD^ BV-2 cells compared with the NC^KD^ BV-2 cells, and this significant increase could be inhibited by the ERK1/2 inhibitor (SCH772984) (Fig. 5A, C). We further assessed the nuclear distribution of TFEB, a master regulator of lysosomal biogenesis. Western blotting revealed that with exposure to PFFs, the NC^KD^ BV-2 cells presented significantly greater expression of nuclear TFEB and lower expression of cytoplasmic TFEB; the TREM2^KD^ BV-2 cells presented significantly lower expression of nuclear TFEB and greater expression of cytoplasmic TFEB, which could be reversed by SCH772984 (Fig. 5B, C). Total TFEB expression remained unchanged across groups. Immunofluorescence also revealed that PFFs exposure increased the nuclear-to-cytoplasmic TFEB fluorescence ratio in NCKD BV-2 cells but reduced it in TREM2KD BV-2 cells, with SCH772984 pretreatment restoring this balance (Fig. 5D, E). These results suggested that TREM2 knockdown disrupts TFEB nuclear localization through ERK1/2 pathway activation.

Then we examined the effects of SCH772984 pretreatment on lysosomal function and asyn degradation. After exposure to PFFs, knockdown of TREM2 exacerbated the significant decrease in DQ-BSA, Lysotracker DR, and CathB fluorescence intensity, indicating impaired lysosomal function, while SCH772984 reversed these declines (Fig. 5F, H). Immunofluorescence revealed that after exposure to PFFs, the α-Syn in the TREM2^KD^ BV-2 cells was significantly increased and the co-localization of α-Syn with Lamp1 was significantly decreased compared with the NC^KD^ BV-2 cells, which were mitigated by SCH772984 (Fig. 5G, I). These results suggested that inhibiting TREM2 knockdown exacerbates α-Syn-induced lysosomal dysfunction by activating the ERK1/2 pathway, which inhibits TFEB nuclear distribution, blocking ERK1/2 activation restores lysosomal function and enhances α-Syn clearance.

### Knockdown of TREM2 in BV-2 cells pretreated with PFFs aggravated α-Syn phosphorylation and apoptosis in HT22 cells

**Fig. 6:**
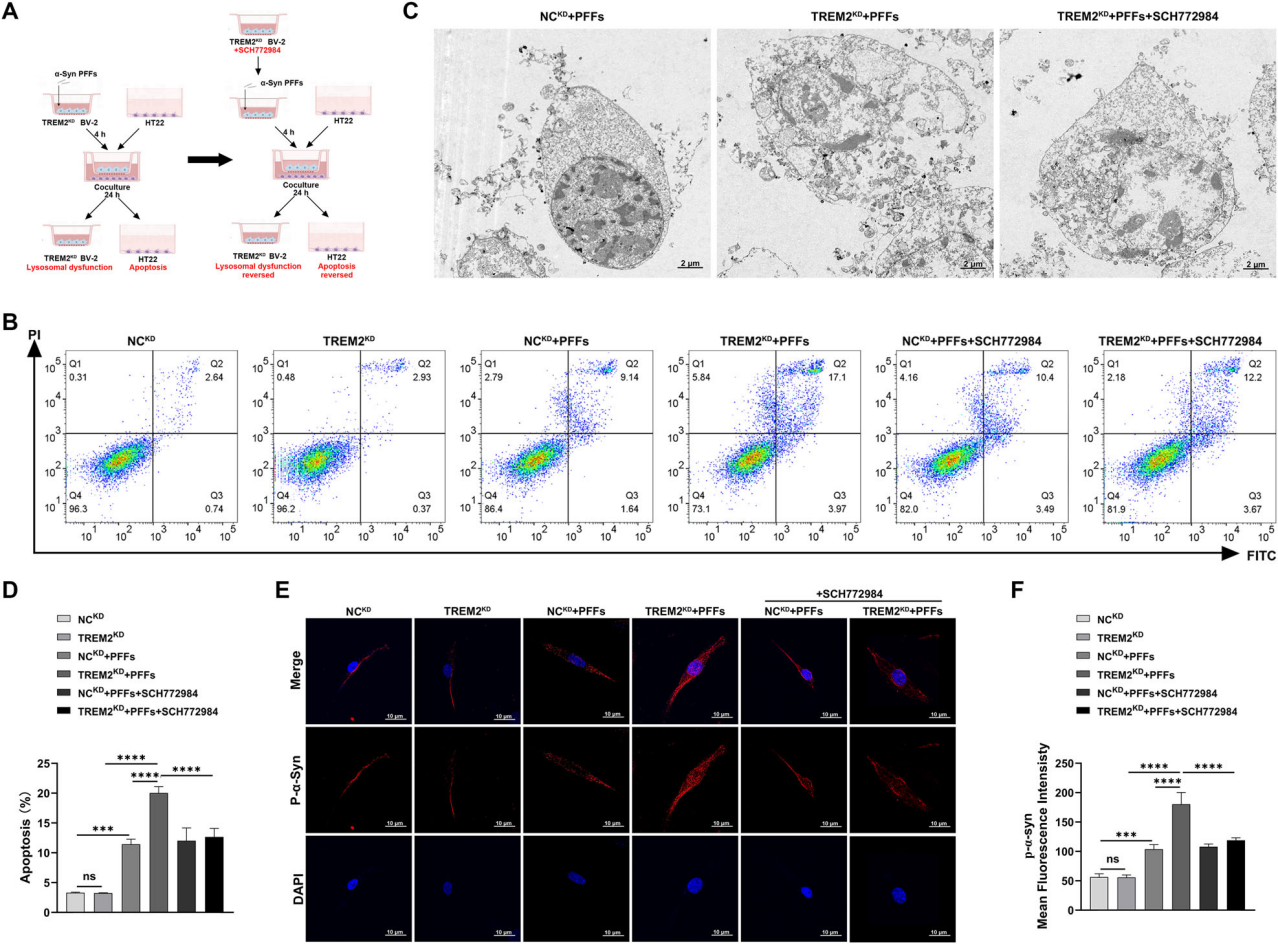
Knockdown of TREM2 aggravated α-Syn phosphorylation and apoptosis in HT22 cells. **A** Coculture of BV-2 cells and HT22 cells. **B** Representative images of flow cytometry of HT22 cells in each group. **C** Representative images of TEM of apoptotic morphology of HT22 cells in each group. **D** Quantification of apoptosis of HT22 cells in each group (*n* = 3, one-way ANOVA and Šídák’s multiple comparisons test). **E** Representative images of p-α-Syn (red) immunofluorescence staining in HT22 cells in each group. Scale bar, 10μm. **F** Quantification of mean Fluorescence intensity of p-α-Syn in HT22 cells in each group (*n* = 3, one-way ANOVA and Šídák’s multiple comparisons test). The error bars represent the ± SDs. ***p < 0.001, ****p < 0.0001. PFFs, recombinant human A53T α-Syn pre-formed fibrils; TEM, transmission electron microscopy.

To examine the impact of TREM2-deficient microglia on neurons, we cocultured PFFs-pretreated BV-2 cells with HT22 neuronal cells. After 4-hour PFFs exposure, BV-2 cells were placed in contact with HT22 cells to mimic microglia-neuron interactions (Fig. 6A). Flow cytometric apoptosis analysis revealed that the apoptosis rates in HT22 cells cocultured with the TREM2^KD^ BV-2 cells and the NC^KD^ BV-2 cells pretreated with PFFs were significantly increased than those without exposing to PFFs, and knocking down TREM2 significantly enhanced the pro-apoptotic ability of BV-2 cells pretreated with PFFs, which could be reversed by SCH772984 (Fig. 6B, D). TEM showed early apoptotic features in HT22 cells cocultured with the PFF-pretreated NC^KD^ BV-2 cells and late apoptotic features with the PFFs-pretreated TREM2^KD^ BV-2 cells, and SCH772984 alleviated the effects of TREM2^KD^ (Fig. 6C).

Immunofluorescence revealed that, in HT22 cells cocultured with the TREM2^KD^ BV-2 cells and the NC^KD^ BV-2 cells pretreated with PFFs, the expression of the p-α-Syn was significantly increased than that without exposing to PFFs, and knocking down TREM2 in BV-2 cells exposing to PFFs significantly increased the expression of the p-α-Syn in HT22 cells, which could be reversed by SCH772984, but showed no change without exposing to PFFs (Fig. 6E, F). As the p-α-Syn increased in the TH22 cells, immunofluorescence showed that the phosphorylation of α-Syn in HT22 cells seemed to be a process of moving from the axon or the end away from the nucleus to the perinuclear region. These results indicated that knockdown of TREM2 in BV-2 cells attenuated the ability to degrade PFFs, leading to enhanced α-Syn phosphorylation and neuronal apoptosis in HT22 cells.

## Discussion

TREM2 has been identified as a neuroprotective factor in multiple diseases, particularly in neurodegenerative conditions[9–14, 40, 41]. In AD, TREM2 inhibits complement-mediated synaptic elimination during neurodegeneration to protect cognitive function [9–14, 40, 41], and acts downstream of CD33, alleviating pathological deposition of amyloid β (Aβ) and improving cognitive function in 5xFAD mice [16]. Moreover, TREM2 deficiency enhances tau spreading to the hippocampus, correlating with impaired synaptic function and memory deficits [13]. Recent studies further highlight TREM2’s protective role in mitigating neurodegeneration and neuroinflammation in PD [17, 18]. Consistent with these findings, we observed significant infiltration of TREM2^+^ microglia in the hippocampus of PD patients and A53T mice. Based on this evidence and the known role of microglia in clearing α-Syn, we hypothesize that TREM2 enhances microglial clearance of misfolded and aggregated α-Syn in the hippocampus, thereby protecting against cognitive impairment. The A53T mice is a common PD mice model that overexpresses the α-Syn mutant A53T in the brain, exhibits widespread α-Syn deposition but develops motor symptoms only after 16 months [42, 43]. This delayed motor symptom onset suggests that prolonged α-Syn burden was required to induce behavioral changes. The vulnerability of hippocampus to the early accumulation of α-Syn has been found in previous studies [38], which may be related to the higher susceptibility of hippocampal neurons to α-Syn [44–46], ultimately leading to the earlier onset of hippocampal neuronal damage. To investigate the effect of TREM2 deficiency on cognitive impairment while avoiding confounding effects from motor deficits, we conducted experiments when the mice were 12 months old. Consistent with previous studies, α-Syn accumulation and partial dopaminergic neuron damage were found in the SNpc, and no motor symptom was detected in the A53T mice as well as the TREM2^-/-^/A53T mice. Notably, TREM2 deficiency aggravates hippocampal neurodegeneration and synaptic plasticity impairment in the A53T mice, resulting in more severe cognitive impairment. There revealed that TREM2 deficiency could amplify or accelerate the α-Syn-induced damage of hippocampal neurons, leading to aggravated cognitive impairment in PD mice.

Misfolded and aggregated α-Syn, a hallmark of PD, initially accumulates in neurons and spreads pathologically, eventually being exposed to microglia following neuronal death [47]. Microglia infiltrate around the misfolded and aggregated α-Syn, internalize and degrade them, and have the highest rate of degrading and cleaning α-Syn [48]. However, TREM2 deficiency impairs microglial clearance of metabolic waste and misfolded proteins [37, 49]. In this study, the TREM2^-/-^/A53T mice exhibited the increased accumulation of α-Syn in hippocampus, and more infiltrating microglia around the α-Syn. In addition, the microglial lysosomal phagocytic activity marker CD68 significantly increased in the hippocampus of the TREM2^-/-^/A53T mice. These results suggested that microglia were more activated, and lysosomal phagocytic activity was increased and was contrary to previous studies [21, 50]. These differences may be due to the fact that a large number of the misfolded and aggregated α-Syn affect the functions of microglia, including the activation of reactive microglial [51], exhaustion of phagocytic function [52], inflammatory activation [53], oxidative stress imbalance [54], and so on. However, it was ultimately shown that the accumulation of α-Syn was increased in the hippocampus of the TREM2^-/-^/A53T mice, that is, the clearance disorder of α-Syn occurred after α-Syn was phagocytosed by microglia. Considering that internalized α-Syn would be immediately delivered to lysosomes for degradation [25], we next focused our study on lysosomes and determined that the clearance disorder of α-Syn was due to the impaired lysosomal degradation function. Results showed that α-Syn reduced the expression of proteases, such as Cathepsin B, and Cathepsin D and disrupts lysosomal acidity, which play vital roles in the degradation of α-Syn in lysosomes [55, 56]. The misfolded and aggregated α-Syn form in the acidified lumens of lysosomes [34], inhibits the activity of lysosomal enzymes [21], rupture lysosomal vesicles, and escape from damaged lysosomes [33, 50]. In brief, while lysosomes degrade α-Syn, α-Syn also impairs lysosomal function, and TREM2 deficiency exacerbates the damage of α-Syn on lysosomal function. Here, we revealed that TREM2 deficiency aggravated α-Syn-induced lysosomal dysfunction, including loss of lysosomal enzyme activity, changes in pH, and decreased activity of multiple proteases.

The downstream regulatory mechanism of TREM2 is extremely complex; generally, TREM2 phosphorylates the adaptor proteins DNAX-activated protein (DAP) 12 (DAP12) and DAP10 and activates intracellular signaling pathways, including spleen tyrosine kinase (Syk) pathway and phosphatidylinositol 3-kinase (PI3K) pathway, thereby regulating multiple biological activities, including survival, phagocytosis, inflammatory response, glycolysis, and mTOR signaling [44, 45, 57]. To explore the specific downstream regulatory mechanism of TREM2, we performed transcriptomic analysis and found that TREM2 regulated ATP synthesis coupled electron transport, and neuroactive ligand-receptor interaction in the hippocampus of PD mice. Previous studies have shown that TREM2 also plays a key role during neurodevelopment, mainly in controlling the microglia-mediated process of supernumerary synapse elimination and further affecting neuronal wiring and brain connections [46, 58]. In this process, TREM2 deficiency disrupts microglial bioenergetics and impairs energy metabolism in the entire mitochondrial metabolism of hippocampal tissue, leading to deep transcriptional rearrangements in hippocampal neurons [59], which also explains why the effects of TREM2 deficiency on the A53T mice first manifest as aggravated cognitive impairment. TREM2 exhibits essential neuroactive ligand-receptor interactions in PD mice. In pathological conditions, TREM2 binds to many ligands released from lesions, making the TREM2 pathway central to sensing damage and limiting its spread [60, 61]. Consistent with previous studies, we found that TREM2 deficiency could positively regulate ERK1/2 activation in the exploration of TREM2 downstream pathways. In the TREM2^-/-^/A53T mice, TREM2 deficiency induced the activation of ERK1/2 through the adaptor protein DAP12, increased the phosphorylation level of ERK1/2, and ERK1/2 phosphorylated TFEB at multiple sites [62], leading to the retention of TFEB in the cytoplasm and inactivation of TFEB [63]. When α-Syn impairs lysosomal function, TFEB nuclear translocation increases the expression of lysosomal-related genes [64], activating lysosomal enzymes, lysosomal membrane proteins [65], and v-ATPase subunits [66], and restoring lysosomal function to a certain extent [67]. TREM2 deficiency leading to TFEB inactivation is likely to be one of the key events that aggravates α-Syn-induced lysosomal dysfunction.

The effect of TREM2 on lysosomal function has been reported across various disease models, yet the conclusions remain inconsistent. Some studies reported significant decreases in lysosomal-related genes following TREM2 knockdown [35, 36], while others observed increases or no significant changes [37]. These discrepancies may arise from differences in the extent of TREM2 knockdown, the cell types used, and the specific disease models employed. In this study, no significant alterations in microglial lysosomal function were detected in either TREM2^-/-^ mice or TREM2^KD^ BV-2 cells. This absence of effect may be attributed to compensatory mechanisms under physiological conditions, which sustain lysosomal function in a stable state. However, when pathogenic α-Syn disrupts lysosomal integrity and impairs lysosomal function, this delicate balance is compromised [20]. A rapid transition from a compensated to a decompensated state of lysosomal stability occurred, resulting in more severe microglial lysosomal dysfunction compared to scenarios where TREM2 is expressed normally. This progression ultimately results in pathological accumulation of α-Syn and neuronal damage. In conclusion, TREM2 deficiency aggravates cognitive impairment by exacerbating α-Syn-induced microglial lysosomal dysfunction via the ERK1/2 signaling pathway. This provides a solid basis for the protective role of TREM2 in PD, suggesting a possible research direction for early intervention and treatment of cognitive impairment in PD.

## Materials and methods

### Human specimens

Human specimens were obtained from the Forensic Identification Center of Southern Medical University (Guangzhou, China). Specifically, PD specimens were collected from deceased individuals diagnosed with PD, while control specimens were randomly collected from age-matched individuals who died of other causes.

### Animal

Wild-type C57BL/6 mice, A53T mice, and TREM2^-/-^ mice were purchased from Beijing V-Solid Biotech Co., Ltd (China). The construction of TREM2^-/-^/A53T mice and the identification of the genotypes of each group of mice are shown in Figure S4. For animal experiments, male C57BL/6 mice, A53T mice, TREM2^-/-^ mice, and TREM2^-/-^/A53T mice (*n* = 9, 3 for western bolting, 3 for pathological testing, 3 for transcriptional analyses) were randomly selected, via a random number generator. All the mice were housed in Guangdong Medical Animal Experimental Center under specific pathogen-free (SPF)-grade conditions (temperature 22 °C ± 2°C, humidity 55% ± 5%, 12 h light/dark cycle).

### Behavioral test

Behavioral tests were performed on mice at 12 months of age. All tests were performed between 10 am and 3 pm by experimenters that blind to subgrouping. On the test day, mice were placed in a dimly illuminated behavioral room and were left undisturbed for at least 1 h before testing. Before test, mice were allowed to acclimate undisturbed in a dimly lit behavioral room for at least 1 h. Results of open filed test, NORT, and MWM test were analyzed via SMART version 3.0 software (Panlab, Spain).

#### Open filed test

Locomotor activity was measured in an open field box (40 cm × 40 cm × 40 cm) with indirect lighting (25-50 lux) to avoid reflections and shadows. The odors on the test box and objects were removed with 75% alcohol before each experiment, and at least 10 min between the two mice. After releasing the mouse, the instrument recorded the overall motor ability of the mice in the next 5 min, including total distance moved, velocity, and center time.

#### NORT

Mice were placed into an open field box (40 cm×40 cm×40 cm) with indirect lighting (25-50 lux) to avoid reflections and shadows. During the habituation phase, the mice were allowed to freely explore an open field box without objects for 10 min. In the familiar phase, two same identical objects were placed in the center of the open field, and mice explored for 5 min. In the testing phase, one of the familiar objects was replaced by a novel one, and mice were allowed to explore for 5 min. The exploration of an object was defined as pointing the nose at the object at a distance of less than 1 cm and/or touching it. To minimize the influence of odor traces, the objects and the open field were cleaned with water between each trial. Mice were placed with their backs to the objects to avoid choice preference in each trial. The difference in time spent exploring the familiar object versus the novel object in the testing phase was recorded and calculated as an index of recognition.

#### MWM test

A circular pool (diameter 140 cm, height 50 cm) was filled with water and maintained at 22 °C ± 2 °C. An escape platform (10 cm diameter) was located in the middle of the third quadrant and 1.5 cm below the water surface. The pool was decorated with spatial cues of four different geometric shapes to help mice recognize directions. In a space exploration test, the mice were trained over 5 consecutive days with four trials per day per mouse. A trial begins by placing mice in the water facing the wall of the pool at one of the starting points. If mice failed to escape within 60 s it is gently conducted to the platform by the experimenter and stays here for 20 s. The escape latency and path to the platform were tracked and recorded. The intertrial interval used is 10 min. After each trial, the mice were dried, and returned to their cage at the end of the session. In the acquisition phase, mice were placed in the opposite quadrant from the target quadrant, and the escape latencies, time spent in the target quadrant, numbers of platform crossings, and swimming routes were recorded.

#### Rotarod test

Prior to the rotarod test, mice were placed on the rotarod instrument for 5 min on consecutive 5 days, training them at a speed of 10 rpm. During the formal experiment, the start speed was adjusted to 4 rpm, the acceleration was set to 0.12 rpm/s, and the maximum speed was set to 40 rpm. The experiment was ended if the mouse fell or walked backwards. The falling latencies were recorded, and each mouse was tested 5 times with at least 30 min between each test.

#### Traction test

The front paws of the mice were placed on a horizontal rope (diameter 5 mm), and the suspension of the limbs of the mice was observed after 10 s. If the mouse grasped the rope with both hind paws, it would be scored 4 points; if the mouse grasped the rope with one hind paw, it would be scored 3 points; if the mouse grasped the rope with both front paws, it would be scored 2 points; and if the mouse grasped the rope with one front paw, it would be scored 1 point.

### Nissl staining

Mice hippocampal tissue sections were Nissl-stained according to the established protocol [68]. Histopathological changes of neurons in the hippocampus were observed under Digital Slide Scanners (Pannoramic MIDI, China), and images were acquired.

### Cell culture and infection

The mouse microglia cell line BV-2 and mouse hippocampal neuron cell line HT22 were purchased from American Type Culture Collection (Manassas, USA). Cells were cultured using Dulbecco’s modified Eagle’s medium (DMEM; Gibco, USA) supplemented with 10% fetal bovine serum (FBS; Gibco, USA) and 1% penicillin-streptomycin (Gibco, USA) and incubated in a humidified incubator at 37 °C with 5% CO_2_. Pre-experimental testing showed no mycoplasma contamination.

Lentivirus expressing shRNA of TREM2 and its corresponding control were purchased from GenePharma (Shanghai, China) [18]. BV-2 cells were infected with Lipofectamine™ 3000 (Invitrogen, USA) to construct a TREM2 knockdown BV-2 stable cell line (TREM2^KD^ BV-2 cells) and its control (NC^KD^ BV-2 cells). To examine the role of pathway of ERK1/2, microglial were treated with ERK1/2 inhibitors (SCH772984, 1uM).

### Preparation and validation of PFFs

The preparation and validation of PFFs were shown in Figure S5, following the established protocol [69] with slight modifications. Briefly, full length human A53T gene in Pet16b vector was transformed in *Esherichia coli* BL21 (DE3) strain (Novagen, Birmingham, United Kingdom). α-Syn monomers were subsequently purified using Hi-Trap Q HP anion exchange column (GE Healthcare, USA) on an Akta purifier (GE Healthcare, USA). The purified α-Syn monomers were validated by running the samples in 12% SDS-PAGE followed by immunoblotting using anti-α-Syn antibody (1:500, Abcam, ab138501). Purified α-Syn monomers were subjected for an Amicon Ultra-0.5-3 kDa cut-off filter (Millipore, USA) in dialysis buffer (phosphate buffer saline, PBS) ultrafiltration for removing salt ions and concentrate to a final concentration of 5 mg/mL. To obtain PFFs, the purified α-Syn monomers were followed by agitation for 7 days (1000 rpm, 37 °C). As previously reported, transmission electron microscopy (TEM) was used to observe aggregate morphology, Thioflavin T (ThT) was used to confirm aggregate formation, and Pierce LAL Chromogenic Endotoxin Quantification Kit (Thermo Fisher Scientific, USA) was used to confirm that endotoxin levels in PFFs were less than 0.02 endotoxin units/ug (EU/μg).

### DQ-BSA assay, LysoTracker Red assay, and CathB activity assay

The BV-2 cells were plated in confocal dishes (1×10^5^ cells/dish). After exposing to PFFs 4 h, DQ-BSA Red assay (1 nM, 12 h) (ShareBio, China), LysoTracker Red (1 nM, 0.5 h) (Beyotime, China), and Magic Red Cathepsin Assay kit (1 nM, 0.5 h) (Immuno-Chemistry Technologies, USA) were used to evaluate lysosomal functions via confocal microscopy (FV3000, OLMPUS, Japan) in live conditions. All images were acquired with the identical settings. For all experiments, the integrated density of DQ-BSA, Losotracker DR, and CathB signal was measured by using ImageJ software (National Institutes of Health, USA) a region of interest was manually created by the freehand selection tool.

### Flow cytometric analysis of apoptosis

Flow cytometry was performed to evaluate the apoptosis of TH22 cells cocultured with BV-2 cells pretreated with PFFs, and the Annexin V-FITC Apoptosis Detection Kit (Beyotime, China) was used according to the manufacturer’s protocol. Blank controls and single-staining controls were set up, and each experiment was performed at least three times. The apoptosis rate of HT22 cells was evaluated via flow cytometry (BD, USA) within 1 h.

### Transmission electron microscopy (TEM)

TEM was performed to observe the apoptotic morphology of TH22 cells cocultured with BV-2 cells pretreated with PFFs, including chromatin changes and apoptotic bodies. After coculture overnight, HT22 cells were collected in electron microscopy fixative (Servicebio, China), fixed, dehydrated, dried, and conductive, and images were subsequently obtained at 80 kV via a Hitachi TEM system (Hitachi, Japan).

### Transcriptional analyses

Transcriptional analyses of hippocampal tissues from A53T mice and TREM2^-/-^/A53T mice were performed by Illumina NovaSeq sequencing system (Biomarker Technologies Corporation, Beijing, China). After verifying the data integrity and validity, differential gene expression analysis was performed by the DESeq package in R software. The GO Gene Ontology (GO) and Kyoto Encyclopedia of Genes and Genomes (KEGG) analysis of DEGs was performed by the hypergeometric distribution test.

### Immunohistochemistry

Immunohistochemistry was performed to determine the expression and localization of tyrosine hydroxylase (TH). The brain tissue samples were routinely into paraffin sections. After dewaxing and rehydration, the brain tissue paraffin sections were repaired with EDTA repair solution (Beyotime, China) in a boiling water bath for 30 min and were blocked with goat serum. The sections were incubated with anti-TH (1:500, Abcam, ab137869) in PBS containing 0.2% Tween-20 (PBST) diluted overnight at 4 °C. Signal detection was performed using the SP Rabbit & Mouse HRP Kit (CW2069, Cowin Biosciences, China), following the manufacturer’s instructions. The sections were then counterstained with hematoxylin, mounted with neutral gum, and covered with a coverslip. After drying, the samples were imaged using a Zeiss META system (Pannoramic MIDI, China) and analyzed with ImageJ software.

### Immunofluorescence

Immunofluorescence was performed to determine the expression and localization of proteins. The human specimen tissues and mouse brain tissues were routinely processed into paraffin sections. After dewaxing and rehydration, the paraffin sections were repaired with EDTA repair solution (Beyotime, China). The human specimen tissue paraffin sections, the mouse brain tissue paraffin sections, BV-2 cells, and HT22 cells were permeabilized and blocked with 0.3% Triton X-100 (Beyotime, China) and 5% BSA (Beyotime, China) in PBS for 2 h at room temperature. The sections or cells were incubated with primary antibodies anti-TREM2 (1:500, Cell Signaling, 91068), anti-TREM2 (1:500, Affinity, DF12529), anti-IBA1 (1:500, Oasisbiofarm, OB-MMS039-01), anti-TH (1:500, Abcam, ab137869), anti-α-Syn (1:500, Abcam, ab138501), anti-p-α-Syn (1:500, Abcam, ab51253), anti-PSD95 (1:500, Abcam, ab238135), anti-synaptophysin (1:500, Abcam, ab32127), anti-NeuN (1:500, Proteintech, 26975-1-AP), anti-CD68 (1:500, Proteintech, 28058-1-AP), anti-CathB (1:500, Abcam, ab214428), anti-Lamp1 (1:500, Abcam, ab278043), anti-TFEB (1:500, Proteintech, 13372-1-AP) in PBST diluted overnight at 4 °C. After being washed with PBST three times, the sections or cells were incubated with AlexaFluor 488-conjugated goat anti-mouse (Thermo Fisher Scientific, A-10680) and AlexaFluor 555-conjugated goat anti-rabbit (Thermo Fisher Scientific, A-21428) secondary antibodies at room temperature in a dark room for 2 h. After washing, the sections or cells were sealed with mounting medium with DAPI (Abcam, ab104139), and fluorescence images were acquired via confocal microscopy (FV3000, OLMPUS, Japan). Photomicrographs were captured and analyzed with FV31S software.

### Western blotting

Western blotting was performed to determine the relative expression of proteins. Total proteins were extracted from cells or mice brain tissues (substantia nigra, hippocampus, and cortex of mice) via a radioimmunoprecipitation assay (RIPA; Beyotime, China) with phenylmethanesulfonylfluoride fluoride (PMSF; Beyotime, China) and a phosphatase inhibitor cocktail (Beyotime, China). Nuclear proteins and cytoplasm proteins were extracted from cells or mice hippocampal tissues via NE-PER™ Nuclear and Cytoplasmic Extraction Reagent (Thermo Fisher Scientific, USA) with PMSF and phosphatase inhibitor cocktail. After ultrasonic crushing and high-speed centrifugation at 4 °C, protein concentrations were measured with a bicinchoninic acid (BCA) protein assay kit (Beyotime, China). 20 μg of proteins were separated via SDS-PAGE and transferred onto polyvinylidene fluoride (PVDF) transfer membranes (Millipore, USA) with a 0.22 μm pore size. After being blocked for 2 h with Tris-buffered saline (TBS) containing 5% nonfat skim milk (Beyotime, China) or BSA (Beyotime, China), the membranes were incubated overnight at 4 °C with the following primary antibodies: anti-TH (1:2000, Abcam, ab137869), anti-α-Syn (1:1000, Abcam, ab138501), anti-p-α-Syn (1:2000, Abcam, ab51253), anti-PSD95 (1:2000, Abcam, ab238135), anti-synaptophysin (1:2000, Abcam, ab32127), anti-NeuN (1:2000, Proteintech, 26975-1-AP), anti-CathB (1:2000, Abcam, ab214428), anti-CathD (1:2000, Abcam, ab75811), anti-Lamp1 (1:2000, Abcam, ab278043), anti-TREM2 (1:2000, Affinity, DF12529); anti-ERK1/2 (1:2000, Abmart, T40071), anti-p-ERK1/2 (1:2000, Abmart, TA1015), anti-TFEB (1:2000, Proteintech, 13372-1-AP), anti-Histone-H3 (1:5000, Abcam, ab1791), anti-β-Tubulin (1:50000, Proteintech, 2146). After being washed with TBST, the membranes were incubated with HRP-conjugated goat anti-rabbit (1:2000, Yamei, LF102) or HRP-conjugated goat anti-mouse (1:2000, Yamei, LF101) secondary antibodies at room temperature for 2 h. After washes, the signals were detected via enhanced chemiluminescence (ECL) reagent (Millipore, USA) and a chemiluminescence system (Tanon, China). Images were analyzed via ImageJ software. Each experiment was repeated at least three times.

### Statistical analyses

All cell experiments and animal sample sizes were predetermined to achieve at least 3 biological replicates without prior calculation of power and any exclusion criteria. The statistical analyses were performed via the GraphPad Prism version 10. 1. 2 (GraphPad Software, USA). Statistical analyses were performed via an unpaired two-tailed Student’s t test for comparing two groups, a one-way analysis of variance (ANOVA) test, or a two-way ANOVA test for comparing more than two groups. Data from replicate experiments are represented as mean ± SD. Statistically significant differences were indicated when *p* < 0.05.

## Supplementary information

Original full length western blots

Supplementary Materials

## Data Availability

The datasets during and/or analyzed during the current study are available from the corresponding author on reasonable request.
