# Supplementary material for: TREM2 deficiency exacerbates cognitive impairment by aggravating α-Synuclein-induced lysosomal dysfunction in Parkinson’s disease
Source: Cell Death Discov. 2025 May 20;11:243. doi: 10.1038/s41420-025-02538-1 (PMC12092616; doi:10.1038/s41420-025-02538-1)
Supplement: Supplementary file 1 — Original full length western blots [file 41420_2025_2538_MOESM1_ESM.docx]

**

 Figure 2 A. β-Tubullin**

**

 Figure 2 A. Oligomer-α-Syn**

**

 Figure 2 A. p-α-Syn**

**
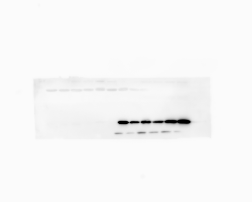
 Figure 2 A.α-Syn**

**

 Figure 2 H. β-Tubullin**

**

 Figure 2 H. CathB**

**

 Figure 2 H. CathD**

**

 Figure 2 H. Lamp1**

**
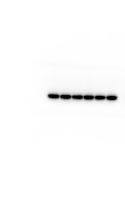
 Figure 5 A. β-Tubullin**

**

 Figure 5 A. TREM2**

**

 Figure 5 A. ERK**

**

 Figure 5 A. p-ERK**

**

 Figure 5 B. Total-β-Tubullin**

**
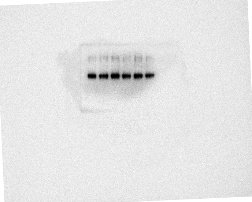
 Figure 5 B. Total-TFEB**

**
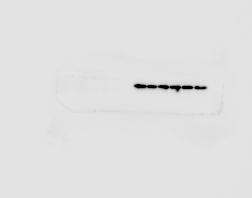
 Figure 5 B. Nuclear-H3**

**

 Figure 5 B. Nuclear- TFEB**

**
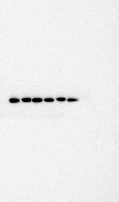
 Figure 5 B. Cytoplasm-β-Tubullin**

**

 Figure 5 B. Cytoplasm-TFEB**



**Figure S1 I. β-Tubullin**

**

 Figure S1 I. TH, Tyrosine Hydroxylase**

**

 Figure S2 A. β-Tubullin**

**
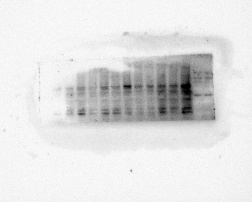
 Figure S2 A. Oligomer-α-Syn**

**

 Figure S2 A. P-α-Syn**

**

 Figure S2 A. α-Syn**

**

Figure S3 E. β-Tubullin**

**

Figure S3 E. NeuN**

**

Figure S3 E.PSD95**

**

Figure S3 E. Synaptophysin**
