# Supplementary material for: TREM2 deficiency exacerbates cognitive impairment by aggravating α-Synuclein-induced lysosomal dysfunction in Parkinson’s disease
Source: Cell Death Discov. 2025 May 20;11:243. doi: 10.1038/s41420-025-02538-1 (PMC12092616; doi:10.1038/s41420-025-02538-1)
Supplement: Supplementary file 2 — Supplementary Materials [file 41420_2025_2538_MOESM2_ESM.docx]

**Table S1. Human specimens in the present study.**

| Group | PD-1 | PD-2 | PD-3 | Control-1 | Control-2 | Control-3 |
| --- | --- | --- | --- | --- | --- | --- |
| Cause of death | Sudden death | Traffic accident | Sudden cardiac death | Traffic accident | Traffic accident | Traffic accident |
| Sex | Male | Male | Male | Male | Male | Male |
| Age (year) | 60 | 58 | 70 | 60 | 58 | 70 |
| Duration (year) | 6 | 5 | 10 | / | / | / |

PD, Parkinson’s disease.


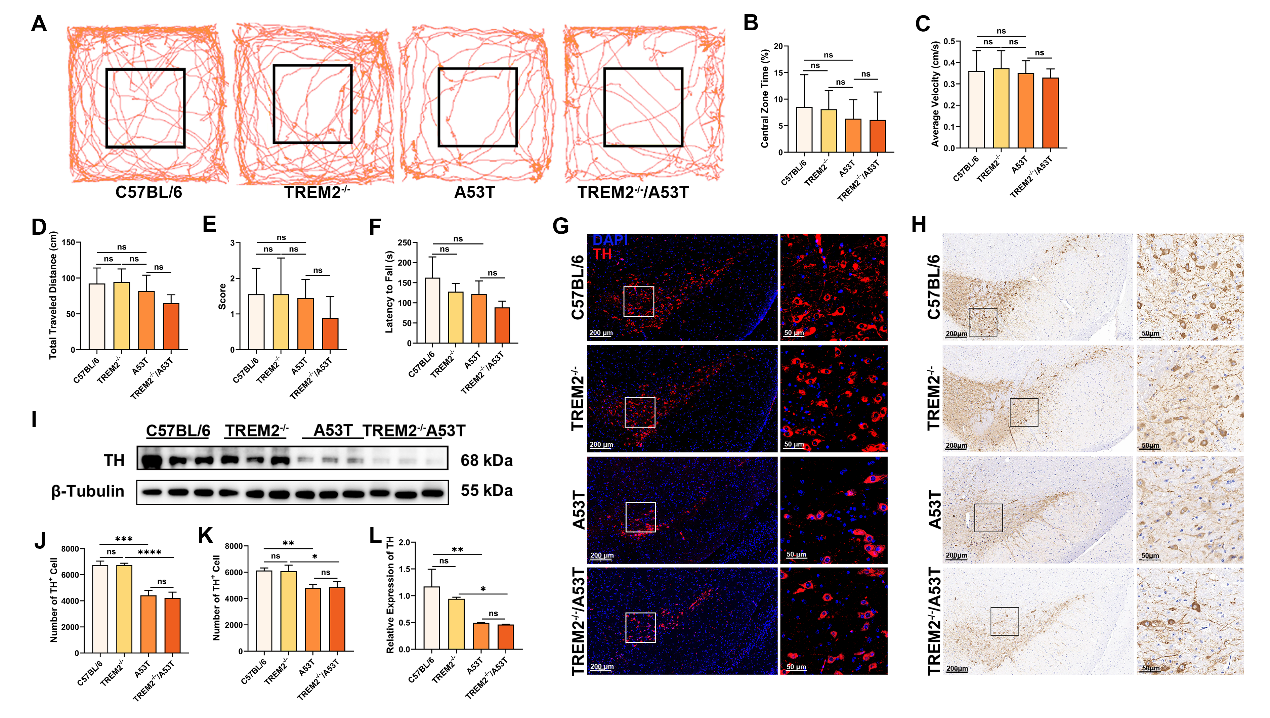


**Figure S1. TREM2 deficiency** **showed no significant effect on motor symptoms.**

**A** Representative image showing the paths obtained from each group in the acquisition phase in Open field test. **B** Central zone time (%), **C** average velocity (cm/s), and **D** total distance traveled (cm) in Open field test (n=9, one-way ANOVA and Šídák's multiple comparisons test). **E** The average score (s) in the traction test (n=9, one-way ANOVA and Šídák's multiple comparisons test). **F** The average latency to fall (s) in rotarod test (n=9, one-way ANOVA and Šídák's multiple comparisons test). **G** Representative images of TH (red) immunofluorescence staining in the SNpc of in each group. Scale bar, 200μm. **H** Representative images of TH immunohistochemical staining in the SNpc of in each group. Scale bar, 200μm. **I** Representative images of western blotting analysis of TH expression in each group. **J** Quantification of number of TH^+^ cells in immunofluorescence staining in the SNpc of in each group (n=3, one-way ANOVA and Šídák's multiple comparisons test). **K** Quantification of number of TH^+^ cells in immunohistochemical staining in the SNpc of in each group (n=3, two-way ANOVA and Tukey's multiple comparisons test). **L** Quantification of TH protein relative expression (n=3, one-way ANOVA and Šídák's multiple comparisons test). The error bars represent the ± SDs. *p<0.05, **p<0.01, ***p<0.001, ****p<0.0001. SNpc, substantia nigra pars compacta; TH, Tyrosine Hydroxylase.

**
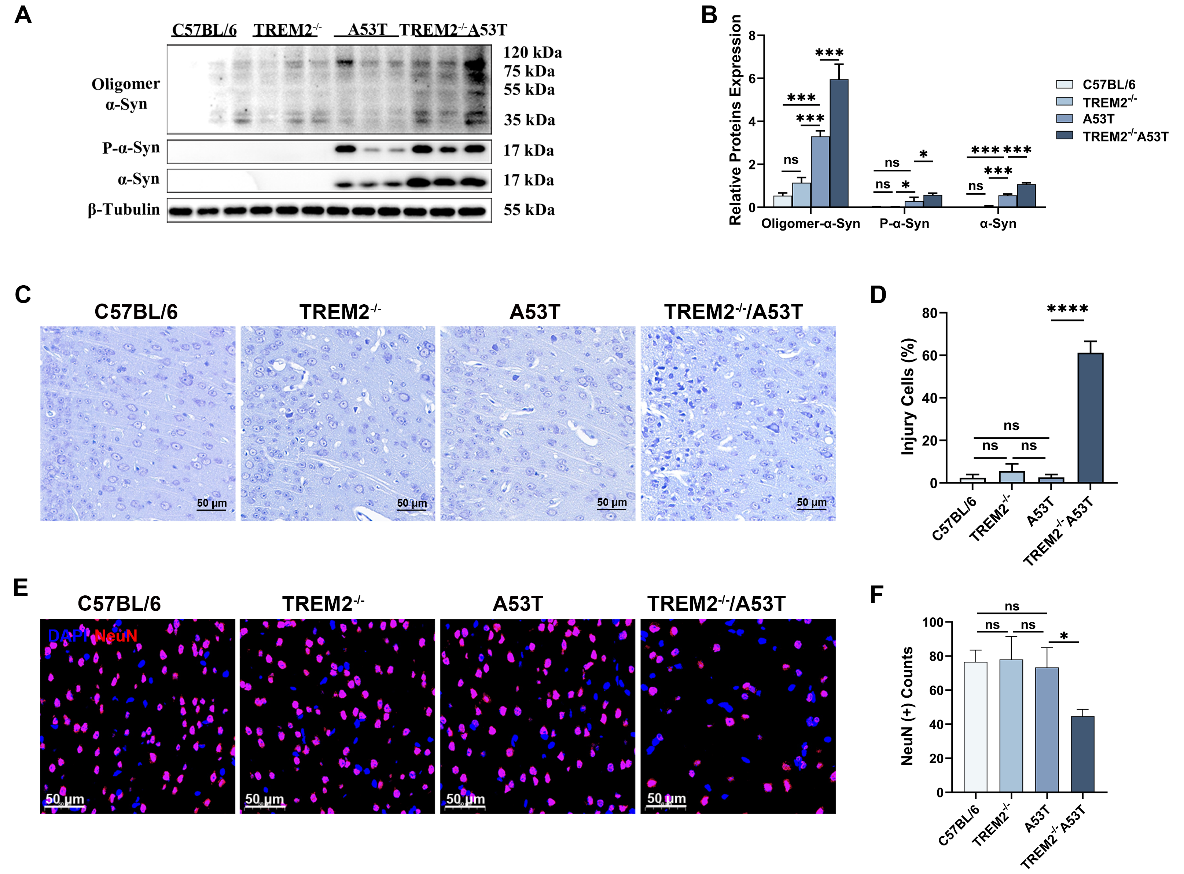
**

**Figure S2. TREM2 deficiency aggravated pathological manifestations in the cortex in A53T mice.**

**A** Representative western blot showing the relative expression of oligomer α-Syn, p-α-Syn, and α-Syn expressions in each group (normalized to β-Tubulin expression). **B** Densitometric analyses of the relative expression of oligomer α-Syn, p-α-Syn, and α-Syn (n=3, one-way ANOVA and Tukey's multiple comparisons test). **C** Representative images of Nissl staining in the cortex of each group. Scale bar, 50 μm. **D** Quantification of the injure neurons in Nissl staining in the cortex of each group (n=3, one-way ANOVA and Tukey's multiple comparisons test). **E** Representative images of NeuN (red) immunofluorescence staining in the cortex of each group. Scale bar, 50 μm. **F** Quantification of number of NeuN^+^ cells in the cortex of each group detected by immunofluorescence (n=3, one-way ANOVA and Tukey's multiple comparisons test). The error bars represent the ±SDs. *p<0.05, ***p<0.001, ****p<0.0001.


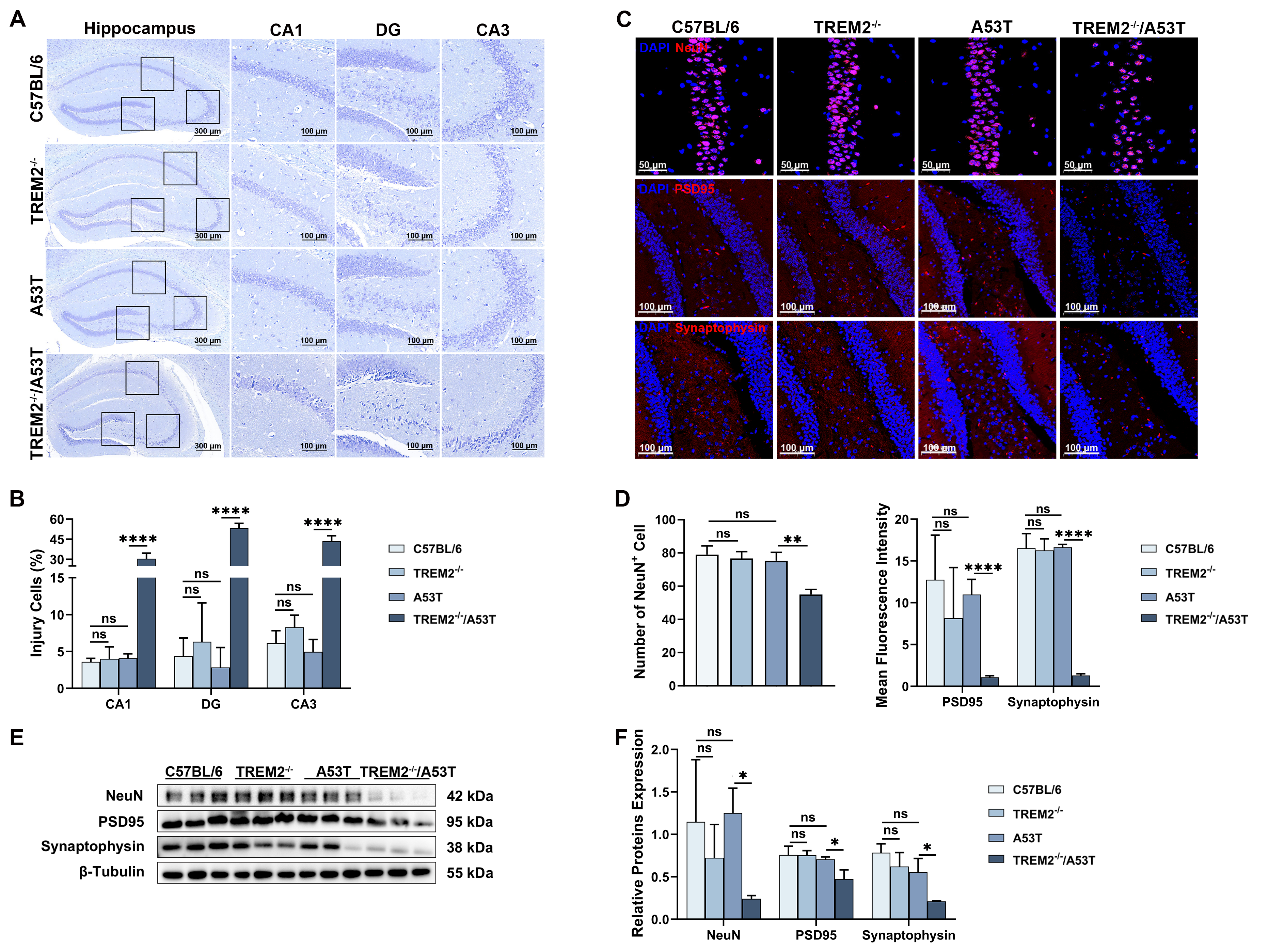


**Figure S2. TREM2 deficiency aggravated pathological manifestations in the hippocampus in A53T mice.**

**A** Representative images of Nissl staining in the hippocampus of each group. Scale bar, 300μm. **B** Quantification of the injure cells (s) in Nissl staining in the hippocampus of each group (n=3, one-way ANOVA and Šídák's multiple comparisons test). **C** Representative images of NeuN (red), PSD95 (red), and Synaptophysin (red) immunofluorescence staining in the hippocampus of each group. Scale bar, 50μm, 100μm, 100μm. **D** Quantification of number of NeuN^+^ cells, mean Fluorescence intensity of PSD95 and Synaptophysin in the hippocampus of each group (n=3, one-way ANOVA and Šídák's multiple comparisons test). **E** Representative images of western blotting analysis of NeuN, PSD95 and Synaptophysin expressions in each group. **F** Quantification of NeuN, PSD95 and Synaptophysin proteins relative expression (n=3, one-way ANOVA and Šídák's multiple comparisons test). The error bars represent the ±SDs. *p<0.05, **p<0.01, ****p<0.0001.


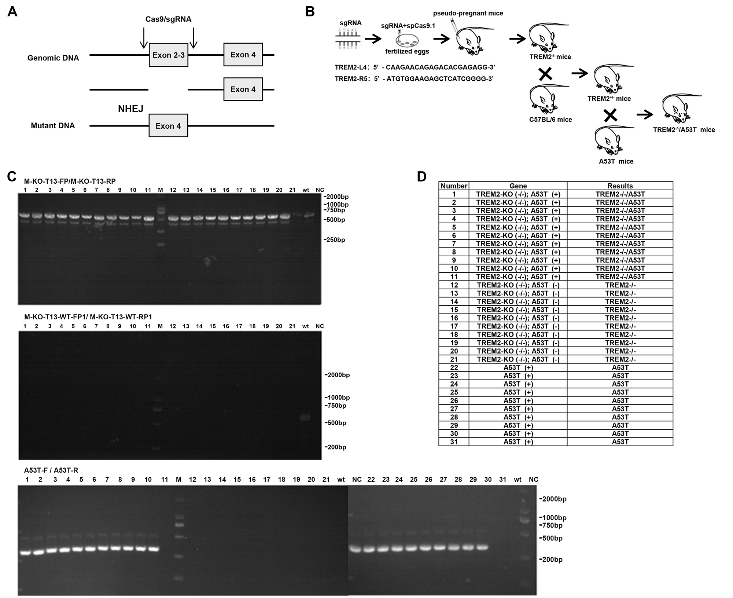


**Figure S4. Experimental mice in the present study.**

**A** Schematic diagram of knocking out the TREM2 gene. **B** The diagram of the TREM2^-/-^/A53T mice construction process. **C** Representative images of agarose electrophoresis of mice in each group. **D** Genotype of mice in each group. TREM2^-/-^/A53T mice, TREM2-deficient A53T mice.


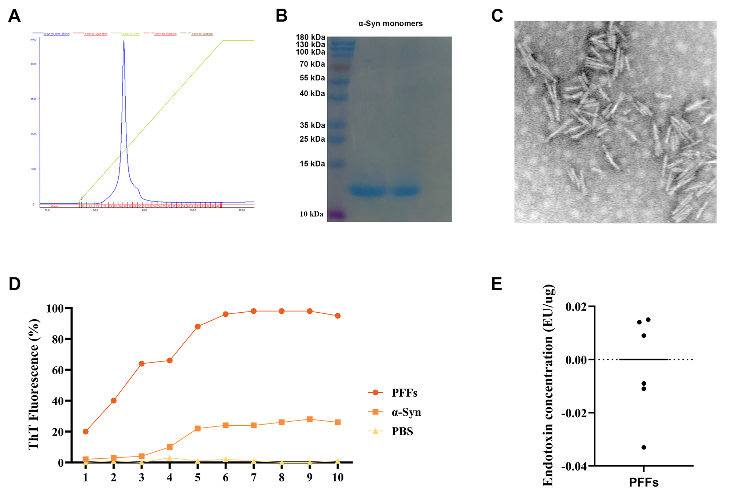


**Figure S5.** **Preparation and validation of PFFs.**

**A** α-Syn monomers were purified by Akta purifier. **B** Representative image of the purified α-Syn monomers was validated by 12% SDS-PAGE. **C** Representative image of TEM of PFFs. **D** Quantification of THT fluorescence (%) fo PFFs. **E** Quantification of endotoxin levels of PFFs. PFFs, recombinant human A53T α-Syn pre-formed fibrils; ThT, Thioflavin T.
